# Supplementary material for: Age-specific contacts and travel patterns in the spatial spread of 2009 H1N1 influenza pandemic
Source: BMC Infect Dis. 2013 Apr 15;13:176. doi: 10.1186/1471-2334-13-176 (PMC3644502; doi:10.1186/1471-2334-13-176)
Supplement: Additional file 1 — Age-specific contacts and travel patterns in the spatial spread of 2009 H1N1 influenza pandemic. [file 1471-2334-13-176-S1.pdf]

Additional File

Age-specific contacts and travel patterns in the spatial spread of 2009 H1N1 influenza pandemic

Andrea Apolloni[1*], Chiara Poletto[2,3,4*], Vittoria Colizza[3,4,5†]

1) Department of Infectious Disease Epidemiology, London School of Hygiene and Tropical Medicine, London, United Kingdom
2) Computational Epidemiology Laboratory, Institute for Scientific Interchange (ISI), Torino, Italy
3) INSERM, U707, Paris, France
4) UPMC Université Paris 06, Faculté de Médecine Pierre et Marie Curie, UMR S 707, Paris, France
5) Institute for Scientific Interchange (ISI), Torino, Italy

*These authors contributed equally to the work
†corresponding author: vittoria.colizza@inserm.fr

Data

European air-transportation network

Here we provide the statistics associated to the European air transportation network [1,2]. Specifically Figure S1 shows the probability distributions of the number of connections k per airport and of the flows of passengers w_{ij} travelling between any pair of linked airports i and j.

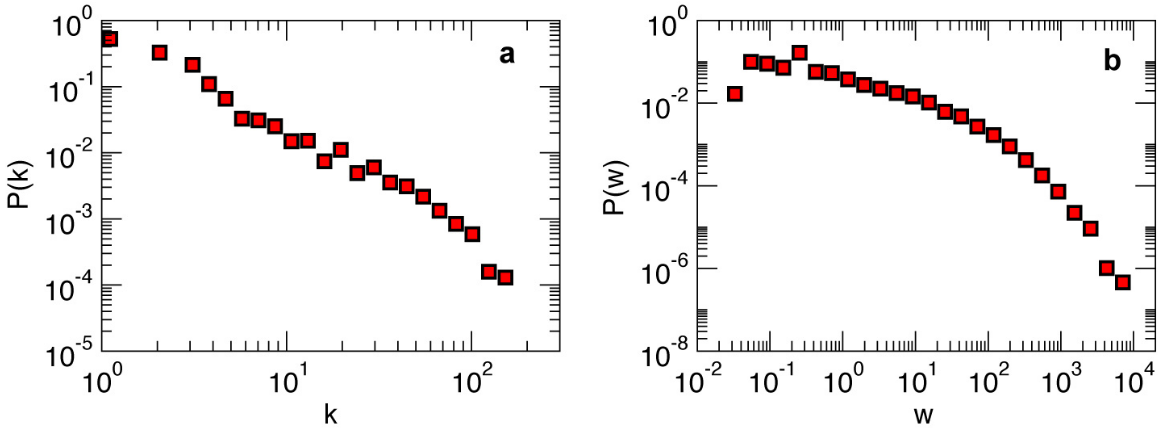

Figure S1: statistics on the European air transportation network. Data source: Eurostat [1], see also Ref. [2]. (a) Distribution of the airport degree k, i.e. airport's number of the flight connections. The Figure clearly shows the power law like behavior characteristic of the distribution. (b) Distribution of the weights w associated to the links: the weight w_{ij} between the pair of linked airports i and j is defined as the average number of people traveling each day between i and j. The quantity is extremely heterogeneous and spans several orders of magnitude.

Sources of travel statistics

In the following we provide the sources of traveling statistics stratified by age presented in Figure 1 and discuss in the main paper:

Helsinki airport:
http://www.lawa.org/uploadedfiles/lax/pdf/2006LAXPassengerSurveyFinal.pdf
Teheran airport: http://amonline.trb.org/20vv77/20vv77/1
London airports (Heathrow, Gatwick, Stansted, Luton):
http://www.londonsdc.org/documents/research/lsdc_airtransportskm.pdf
Amsterdam airports:
http://www.schiphol.nl/B2B/Advertising/MediaProducts/AirportDemographics.htm
Venice airport: http://tesi.cab.unipd.it/142/1/Mazzetto.pdf
German airports (Hannover, Frankfurt, Hamburg, Munich):
http://www.mediafrankfurt.biz/en/infopool/passengerprofile/index.php?block=3
http://www.media-frankfurt.de/1126.html?&no_cache=1&L=1

Age classes

Population estimates by age were obtained ffrom the following sources: Eurostat [1] for the eight Polymod countries with age brackets of 1 year; U.N. data [3] for Mexico, with age brackets of 5 years; US Census [4] for the US, with age brackets of 1 year. Indicating with n the total number of age classes in the dataset, we have $n = 66$ for Europe, $n = 7$ for Mexico and $n = 91$ for the US.

The age grouping used in the specific data set constraints our definition for children and adults classes. For European countries and the US we define children the group of individuals below 18 years. For Mexico, children are individuals below 15 years. We indicate with n_c the number of children age classes. Indicating with N_i the size of age class i, then the fraction of children in each country is evaluated as:

$$\alpha = \frac{\sum_{i=1}^{n_c} N_i}{\sum_{i=1}^{n} N_i} \qquad \text{(S1)}$$

The fraction of children in Europe corresponds to the weighted average over all countries.

Calculation of the contacts matrices

We describe in the following the procedure adopted for evaluating the parameters η, ε from the Mexican and the eight Polymod countries' contact matrices. For the United States case, since we have used individual synthetic information on contacts, the procedure will be described in the corresponding section.

The elements of the contact matrices are estimates of the average number of contacts established by an individual belonging to group i with individuals in group j, and we indicate with c_{ij}. While contact data for the European countries are obtained through contact diary surveys [5], for the Mexican case an “ad hoc model” has been implemented to be fitted against epidemiological data [6]. The procedure adopted to build up the two groups contact matrices involves two steps: *simmetrization* to get rid of possible respondent bias; *aggregation* to evaluate the parameters η, ε.

Data collected are in general asymmetric, i.e. they don’t satisfy the relation $c_{ij}N_i = c_{ji}N_j$, due to the fact that respondents can overestimate or underestimate their social interactions. We correct this error by assuming inter-group contacts being the average between the two values, namely we assume

$$c_{ij} = \frac{1}{2}\frac{c_{ij}N_i + c_{ji}N_j}{N_i} \text{ for } i \neq j \quad \text{(S2)}$$

This operation is repeated for all group pairs *i* and *j*.

Once symmetrized, we start from the elements c_{ij} to evaluate the parameters η,ε of the 2x2 contact matrix describing interactions among children and adults. We first evaluate the average number of contacts of children and adults, q_c and q_a respectively by taking the weighted averages, over the corresponding age classes, of the total number of contacts made by of an individual of that specific class. The average number of contacts established by an individual in the age group i is the sum of the elements of the row i, therefore

$$q_c = \frac{\sum_{i=1}^{n_c}\sum_{j=1}^{n} c_{ij}N_i}{\sum_{i=1}^{n_c} N_i} \quad q_a = \frac{\sum_{i=n_c+1}^{n}\sum_{j=1}^{n} c_{ij}N_i}{\sum_{i=n_c+1}^{n} N_i} \quad \text{(S3)}$$

The parameter η is then evaluated as:

$$\eta = \frac{q_a}{q_c} \quad \text{(S4)}$$

The parameters $\varepsilon_a, \varepsilon_c$, representing the fraction of contacts established outside groups, are computed as

$$\varepsilon_c = \frac{1}{q_c}\frac{\sum_{i=1}^{n_c}\sum_{j=n_c+1}^{n} c_{ij}N_i}{\sum_{i=1}^{n_c} N_i} \quad \varepsilon_a = \frac{1}{q_a}\frac{\sum_{i=n_c+1}^{n}\sum_{j=1}^{n_c} c_{ij}N_i}{\sum_{i=n_c+1}^{n} N_i} \tag{S5}$$

Where the second sum is restricted to contacts with groups not belonging to the class considered. The cross group mixing ε is then evaluated from the symmetry relation on the off diagonal elements of the contact matrix:

$$\varepsilon = \varepsilon_c \alpha = \varepsilon_a \eta (1-\alpha). \tag{S6}$$

Global invasion threshold

Full calculation of R_* expression

In this section we provide the details on the derivation of the global invasion threshold parameter R_*. We recall equation (5) reported in the main paper

$$D_k^n = \sum_{k'} \Omega(\lambda_{kk',a}, \lambda_{kk',c})(k'-1)P(k \mid k')D_{k'}^{n-1}\left(1 - \sum_{m=0}^{n-1}\frac{D_k^m}{V_k}\right) \tag{S7}$$

The term $\Omega(\lambda_{kk',a}, \lambda_{kk',c})$ is the probability that a major epidemic will be triggered in the not-yet-infected population of degree k , by λ_c children and λ_a adults traveling from a diseased subpopulation k'. The term can be written as

$$\begin{aligned}\Omega(\lambda_{kk',a}, \lambda_{kk',c}) &= 1 - \pi_a^{\lambda_{kk',a}} \cdot \pi_c^{\lambda_{kk',c}} \approx (1-\pi_c)\lambda_{kk',c} + (1-\pi_a)\lambda_{kk',a} = \\ &\left\{(1-\pi_c) r z_c + (1-\pi_c)(1-r) z_a\right\}\frac{w_0}{\mu}(kk')^{\theta}\end{aligned} \tag{S8}$$

where the latter approximation is valid in the assumption of mild epidemics, i.e. in the limit of R_0 close to 1, and thus $\pi_c, \pi_a \to 1$. We plug expression (S8) into (S7) in order to re-write the invasion equation in terms of the attack rates, z_c and z_a and of the extinction probabilities, π_a and π_c. We consider the case of uncorrelated networks in which the conditional probability does not depend on the originating node, i.e. $P(k|k') = kP(k)/\langle k\rangle$, and assume that at the early stage of the spatial invasion the number of infected subpopulations can be neglected $\sum_{m=0}^{n-1} \frac{D_k^m}{V_k} \ll 1$. Equation (S7) becomes then

$$\begin{aligned} D_k^n &\approx \sum_{k'} \left\{(1-\pi_c) r z_c + (1-\pi_a)(1-r) z_a\right\} \frac{w_0}{\mu} (kk')^\theta (k'-1) \frac{kP(k)}{\langle k\rangle} D_{k'}^{n-1} \\ &= \left\{(1-\pi_c) r z_c + (1-\pi_a)(1-r) z_a\right\} \frac{w_0}{\mu} \frac{k^{1+\theta} P(k)}{\langle k\rangle} \sum_{k'} (k')^\theta (k'-1) D_{k'}^{n-1} \end{aligned} \quad \text{(S9)}$$

Multiplying both terms by $k^\theta (k-1)$ and summing over k we obtain the recursive equation [7,8]

$$\Theta^n = R_* \Theta^{n-1} \quad \text{(S10)}$$

where $\Theta^n = \sum_k k^\theta (k-1) D_k^n$ and the invasion threshold parameter R_*:

$$R_* = \left\{(1-\pi_c) r z_c + (1-\pi_a)(1-r) z_a\right\} \frac{w_0}{\mu} \frac{\langle k^{2+2\theta}\rangle - \langle k^{1+2\theta}\rangle}{\langle k\rangle}. \quad \text{(S11)}$$

The number of infected subpopulations increases if the threshold condition $R_* > 1$ is satisfied.

The plots of R_* shown in the main paper are obtained by computing the expression (S11) after evaluating numerically z_c, z_a, π_c, π_a. The term $\chi = \frac{\langle k^{2+2\theta}\rangle - \langle k^{1+2\theta}\rangle}{\langle k\rangle}$ is computed for a finite network having $V = 10^4$ nodes and power law degree distribution $P(k) = k^{-\gamma}$, where the two values $\gamma = 3$ and $\gamma = 2$ are considered. Degree takes values in the range $[k_{\min}, k_{\max}]$, where we set $k_{\min} = 1$ and $k_{\min} = 2$ for the two cases $\gamma = 2$ and

$\gamma = 3$ respectively, and $k_{\max} = \sqrt{V}$ for both the degree distributions, as indicated in [9]. Defining the degree interval in such a way ensures that the two degree distribution have the same average degree.

Explicit expression in the limit cases $\eta \to 0$ and $\eta \to 1$

Approximate analytical expression of R_* can be obtained under the assumption $\epsilon \to 0$ in two limit cases: $\eta \to 0$, i.e. adults make very few contacts, and $\eta \to 1$, i.e. the system is homogeneous in terms of average number of contacts. We recall, however, that η assumes also values larger than 1 like the case of Belgium. Another regime of possible interest is given by $\eta >> 1$, however in this case adults would become the main driving force of both the local transmission dynamics and the spatial dissemination of the pathogen, thus leading to an expected trivial decrease of the global threshold condition.

Let's first recall that in taking the two-fold limit $\epsilon \to 0$ and $\eta \to 0$ the relation $\varepsilon < \eta(1-\alpha)$ has to be satisfied in order the model to be consistent. In the limiting cases, $\eta \to 0, \eta \to 1$, we can write the attack rate for children and adults by writing the series expansion around $\epsilon = 0$ and retaining the linear terms either in η either in ε:

$$
\begin{aligned}
z_c &\approx 2\frac{R_0 - 1}{R_0^2} \\
z_a &\approx 2\frac{\varepsilon}{\alpha}\frac{R_0 - 1}{R_0}\left(1 + R_0^{-1}\eta\right)
\end{aligned}
\qquad \text{(S12)}
$$

for the case $\eta \to 0$, and

$$
\begin{aligned}
z_c &\approx 2\left(\frac{R_0 - 1}{R_0^2}\right) - \varepsilon\frac{2\left(R_0^2 - 2R_0 + 2\right)}{(1-\alpha)\left(R_0 - 1\right)R_0^3}\left(1 - R_0 + (2 - R_0)(1-\eta)\right) \\
z_a &\approx 2\left(\frac{R_0 - 1}{R_0^2}\right) + \frac{2}{R_0^2}\left(3 - 2R_0\right)\left(1-\eta\right) + 2\varepsilon\left(\frac{2}{\alpha R_0^3} - \frac{2}{(1-\alpha)R_0^3} - \frac{1}{R_0^2(1-\alpha)}\right)
\end{aligned}
\qquad \text{(S13)}
$$

for the case $\eta \to 1$.

We notice that, in the case $\eta \to 0$, the fraction of adults infected is sub-leading with respect to children. In the second case, instead, the fractions of infected in both classes are comparable.

With the analogous series expansion the extinction probabilities become:

$$\pi_c \approx \frac{1}{1+R_0\left(1-\pi_c\right)+\frac{R_0\varepsilon}{\alpha}\left(1-\pi_a\right)} \approx \frac{1}{R_0}$$

$$\pi_a \approx \frac{1}{1+R_0\frac{\varepsilon}{(1-\alpha)}\left(1-\pi_c\right)+R_0\left(\eta-\frac{\varepsilon}{(1-\alpha)}\right)\left(1-\pi_a\right)} \approx 1-\varepsilon\left(\frac{R_0-1}{1-\alpha}\right)(1+R_0\eta) \quad \text{(S14)}$$

for $\eta \to 0$, and

$$\pi_C \approx \frac{1}{R_0}\left(1+\frac{\varepsilon}{\alpha}\frac{(R_0\eta-1)}{(R_0-1)\eta}\right)$$

$$\pi_A \approx \frac{1}{R_0}\left(\left(2-\eta\right)+\frac{\varepsilon}{(1-\alpha)}\frac{(1-\eta)}{(R_0-1)}\right) \quad \text{(S15)}$$

for $\eta \to 1$.

In the first case, due to the few contacts established by adults, the epidemic is most likely to die immediately when seeded by adults. In the other case, instead, the probability of triggering the epidemic slightly depends on the type of seed.

The expressions for the attack rate and the extinction probability can be combined providing the explicit form for the invasion threshold parameter R_*. In the case that only adults travel, this reads as

$$R_* = 2\frac{\left(R_0-1\right)^2}{R_0^2}\frac{w_0}{\mu}\chi F(R_0,\alpha,\varepsilon,\eta),$$

where $F(R_0,\alpha,\varepsilon,\eta)$ results in the two limit cases as:

Case $\eta \to 0$	Case $\eta \to 1$	
$\frac{\varepsilon^2}{\alpha\left(1-\alpha\right)}\left(R_0+\left(1+R_0^2\right)\eta\right)$	$1-\varepsilon\frac{2+\left(R_0^2-2R_0-4\right)\alpha}{(R_0-1)R_0^2(1-\alpha)\alpha}+3\left(1-\eta\right)$	(S16)

The factor χ accounts for the heterogeneity of the network: $\frac{\left\langle k^{2+2\theta}\right\rangle-\left\langle k^{1+2\theta}\right\rangle}{\langle k\rangle}$

Table S1 addresses the comparison between the two approximate expressions of R_* and the solution obtained numerically for the cases $R_0 = 1.05,\ 1.20,\ 1.40$ with α set to the European average value. Parameters ε and η are chosen in order to be close to the limit values, thus $\varepsilon = 0.001$ and the two extreme cases 0.01 and 0.99 are considered for η . In order to compare the approximate $R_*(\text{approx.})$ and the numerical R_* values

we measure the relative difference

$$\text{Deviation}(\%)=100\cdot\frac{\left(R_*-R_*(\text{approx.})\right)}{\left(R_*-1\right)}$$

A large value of this quantity can indicate that the approximation is not good enough to determine if the system is over or under the threshold condition. The approximate solutions are in good agreement with the corresponding numerical solutions, in particular in the limit for $\eta\to 0$; larger discrepancies are obtained in the limit $\eta\to 1$ and they are found to decrease in decreasing R_0. The largest deviation corresponds to the case of mild influenza, when the solutions for are more sensible to small variations of the parameters η,ε A more detailed analysis of this comparison is the object of future work [10].

	Small η (η=0.01)			Large η (η=0.99)		
R_0	R_*	$R_*(\text{approx.})$	Deviation (%)	R_*	$R_*(\text{approx.})$	Deviation (%)
1.05	$6.90\cdot 10^{-6}$	$1.48\cdot 10^{-5}$	$8.00\cdot 10^{-4}$	1.73	1.92	26.00
1.20	$7.01\cdot 10^{-5}$	$2.06\cdot 10^{-4}$	$1.40\cdot 10^{-2}$	23.74	26.60	12.52
1.40	$2.45\cdot 10^{-4}$	$2.45\cdot 10^{-4}$	$4.60\cdot 10^{-2}$	68.21	79.56	16.90

Table S 1 Comparison between invasion threshold obtained numerically (R_*), and the approximated results $R_*(\text{approx.})$, for different values of R_0. The difference in estimation is compared to the gap between the theoretical value and the threshold value 1 (Deviation(%)). For each value of R_0 we have considered α fixed to the European average value, and $\varepsilon=0.001$ and η assuming two values: $\eta=0.01$ and $\eta=0.99$.

Calculation of R_* in the case of an SEIR dynamics

The next generation matrix depends on the contact pattern, the transmissibility (β) and the infectious period (μ^{-1}) and doesn't change after the introduction of a latent period (τ^{-1}). The final sizes in the two classes (z_c and z_a) and the extinction probabilities (π_c and π_a) for the SEIR model are then the same as in the SIR case, given the same

transmissibility, recovery rate, and contact patterns. The neat effect of the latency period consists in the delay of the epidemic unfolding, making more efficient the epidemic spread from one subpopulation to another because individuals who contract the disease have more time to travel while epidemiologically active. This is encoded in the general expression of the term $\Omega(\lambda_{kk',a},\lambda_{kk',c})$ that accounts for exposed and infectious stages as

$$\Omega(\lambda_{kk',a},\lambda_{kk',c}) = \{(1-\pi_c)rz_c + (1-\pi_a)(1-r)z_a\}w_0T_G(kk')^{\theta},$$

where $T_G = \frac{1}{\mu} + \frac{1}{\tau}$ is the generation time, i.e. the sum of the infection duration, μ^{-1}, and the latency period, indicated here with τ^{-1}. The rest of Equation (S7) remains unchanged, thus the resulting expression for R_* is given by:

$$R_* = [(1-\pi_c)rz_c + (1-\pi_a)(1-r)z_a]w_0T_G\frac{\langle k^{2+2\theta}\rangle - \langle k^{1+2\theta}\rangle}{\langle k\rangle}.$$

Figure S2 shows R_* as a function of the contact ratio η for the case of Europe, comparing the SIR and SEIR case, with different generation times considered. The red and the green curves compare two situations with the same generation time but different repartitions in infectious and exposed period and show that two cases have the same invasion threshold parameter. By keeping fixed $\mu^{-1} = 2.5$ days, the addition of an exposed period shifts the curve upward.

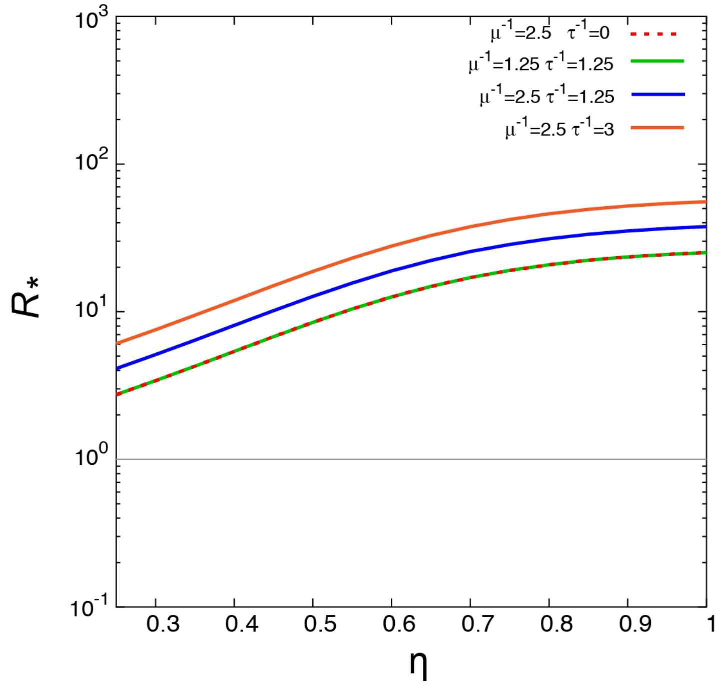

Figure S2: Effect of latency period on the global invasion threshold. R_* as a function of the contact ratio η for the case of Europe. Different generation times are compared. R_* increases linearly with the generation time. The case considered here has $R_0 = 1.2$, mobility air network structure having $P(k) \propto k^{-\gamma}$ with $\gamma = 3$, and contact parameters α and ε set to the European average.

Global invasion threshold for the case of United States

We evaluate the contact structure parameters for the US case by relying on contact network information reconstructed from the synthetic population of Portland and freely available at N.D.S.S.L. website [11]. In the synthetic population approach individuals are endowed with realistic demographic characteristics, drawn from census, as well as activity routines as drawn from mobility surveys: individuals sharing the same location at the same time are assumed to be able to establish links. This approach provides a realistic contact network in Portland [12] evolving in time according to activity patterns. Population is divided in two groups, based on age, and the number of contacts for each individual is recorded thus evaluating the average number of contacts for each group member and how they are distributed. In this way, parameters estimated from this dataset

read as: cross-group mixing $\varepsilon = 0.11$, and contact ratio $\eta = 1.1$. The former is in the ballpark for the estimates for Europe and quite close to the average European value of 0.097, the latter points out a behavior close to the one observed for Belgium, where adults are the principal responsible for the long range seeding and also the drivers of the local spreading since they interact more than children. From census data we have an estimate of the fraction of children $\alpha = 0.24$, which is quite close to the European value.

Figure S3 addresses the comparison between Europe and the US by displaying the global invasion threshold as a function of the contact ratio η for the two cases, and the other parameters (α, ε) fixed to the estimated values. The two curves are almost overlapping which means that the small differences in the estimated ε and α do not impact R_*. Also the difference in η (0.79 of Europe and 1.1 of US) has a small effect, since for higher values of η ($\eta \gtrsim 0.8$) the curve saturates around $R_* \cong 2$.

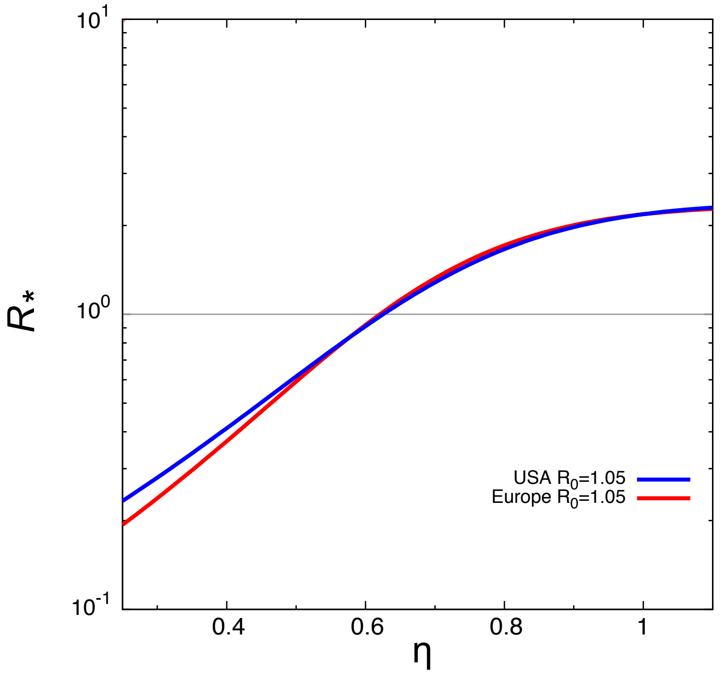

Figure S3: Global invasion threshold as a function of the contact ratio η: comparison between Europe and US. The curves blue and red are obtained by setting the parameter α and ε to the case of the US and Europe respectively. The case considered here has $R_0 = 1.05$ and mobility air network structure having $P(k) \propto k^{-\gamma}$ with $\gamma = 3$.

References

1. Eurostat, http://epp.eurostat.ec.europa.eu/portal/page/portal/eurostat/home/
2. Poletto C, Tizzoni M, Colizza V: **Heterogeneous length of stay of hosts' movements and spatial epidemic spread.** *Nature Scientific Reports* 2012, **2:** 476.
3. **United Nations, Department of Economics and Social Affairs, Population Division, Population Estimates and Projections Sections,** **[**http://esa.un.org/unpd/wpp/Excel-Data/population.htm]
4. **United States of America Census Bureau** **[**http://www.census.gov/2010census/data**]**
5. Goeyvaerts N, Hens N, Aerts M, Beutels P: **Model structure analysis to estimate basic immunological processes and maternal risk for parvovirus B19.** *Biostatistics* 2011, **12**(2):283-302.
6. Fraser C, Donnelly CA, Cauchemez S et al: **Pandemic potential of a strain of influenza A (H1N1): early findings.** *Science* 2009 , **324**: 1557–1561.
7. Colizza V, Vespignani A: **Invasion threshold in heterogeneous metapopulation networks.** *Phys. Rev. Lett.* 2007, **99**: 148701.
8. Colizza V, Vespignani A: **Epidemic modelling in metapopulation systems with heterogeneous coupling pattern: Theory and simulations.** *J. Theor. Biol.* 2008, **251**: 450–467.
9. Catanzaro M, Bogunã M, Pastor-Satorras R: **Generation of uncorrelated random scale-free networks.** *Phys. Rev. E* 2005,**71**: 027103.
10. Apolloni A, Poletto C, Ramasco JJ, Jensen P, Colizza V, in preparation.
11. NDSSL Synthetic data http://ndssl.vbi.vt.edu/opendata/
12. Del Valle SY, Hyman JM, Hethcote HW, Eubank SG: **Mixing patterns between age groups in social networks.** *Social Networks* 2007, **29**: 539–554.
